# The Safety and Efficacy of Hypertonic Saline in Achieving Primary Fascial Closure Following Damage Control Laparotomy: A Systematic Review and Meta-Analysis

**DOI:** 10.7759/cureus.70583

**Published:** 2024-09-30

**Authors:** Neil Muscat, Shaneel Shah, Neill Zammit

**Affiliations:** 1 Vascular Surgery, Manchester Royal Infirmary, Manchester, GBR; 2 General Surgery, Manchester Foundation Trust, Manchester, GBR; 3 General Surgery, University of Malta, Malta, MLT

**Keywords:** 3% sodium chloride hypertonic saline (hts), acute hypernatremia, damage control laparotomy, elevated creatinine, impaired renal function, laparotomy, primary fascial closure

## Abstract

Effective fluid management is critical in patients undergoing damage control laparotomy (DCL) for trauma and sepsis. Hypertonic saline (HTS) has been proposed as an alternative to isotonic fluids to enhance primary fascial closure rates and optimize fluid balance. This systematic review and meta-analysis aims to evaluate the efficacy and safety of HTS compared to isotonic fluids in patients undergoing DCL.

A comprehensive literature search was conducted across multiple databases up to the 14^th^ of June 2024, identifying studies that compared HTS to isotonic fluids in adult patients undergoing DCL for trauma or sepsis. Eligible studies included randomized controlled trials and observational studies reporting outcomes such as early primary fascial closure (EPFC) rates, time to fascial closure, fluid requirements, electrolyte imbalances, renal function, and mortality. Data extraction and quality assessment were performed independently by two reviewers, and pooled analyses were conducted using fixed-effect models where appropriate.

Four studies encompassing 375 patients met the inclusion criteria, with 100 patients receiving HTS and 275 receiving isotonic fluids. HTS administration was associated with a significantly higher EPFC rate compared to isotonic fluids (odds ratio (OR): 0.314; 95% confidence interval (CI): 0.142-0.696; p=0.004). The mean time to fascial closure was also significantly reduced in the HTS group by approximately eight hours (mean difference (MD): 8.007 hours; 95% CI: 5.558-10.596; p<0.001). Patients receiving HTS required significantly less total fluid over 48 hours (MD: 1.055 liters; 95% CI: 0.713-1.398; p<0.001). While HTS use led to higher peak sodium levels (MD: -4.318 mEq/L; 95% CI: -4.702 to -3.934; p<0.001), there were no significant differences in peak creatinine levels, need for inpatient renal replacement therapy, or 28-day mortality between the groups.

HTS appears to be effective in improving EPFC rates and reducing both time to closure and overall fluid requirements in patients undergoing DCL for trauma and sepsis. Although associated with higher serum sodium levels, HTS did not increase the risk of renal dysfunction or mortality. These findings suggest that HTS is a safe and efficacious alternative to isotonic fluids in the management of critically ill patients requiring DCL. Further large-scale, randomized controlled trials are warranted to confirm these results and inform clinical guidelines.

## Introduction and background

Emergency laparotomy is a critical surgical procedure often performed in cases of severe abdominal trauma or sepsis, aiming to diagnose and manage life-threatening intra-abdominal conditions. Emergency laparotomies account for approximately 175,000 procedures annually in the US and 22,000 procedures annually in England and Wales [1].

In the management of severe abdominal trauma, one expeditive approach is through damage control laparotomy (DCL), in which an abbreviated surgery is performed to minimize blood loss and contamination without delay until physiological stability. This process begins with an initial operation for hemodynamic stability, then temporary abdominal closure (TAC), and lastly definitive repair. Its challenges, such as primary fascial closure and large volume fluid shifts, are difficult to manage [2]. Due to its inherent hyperosmolarity, hypertonic saline (HS) has been subject to some investigation as an alternative to normal saline (NS) in the management of trauma patients. HS is believed to have advantages such as improved fluid dynamics control, decreased volume requirement, and increased fascial closure rates; on the other hand, direct application poses significant risks that need further research [3].

HS works primarily through elevating plasma osmolarity, drawing in water from the intracellular compartment into the intravascular compartment, thereby increasing blood volume and mimicking a positive intravascular fluid balance. This is particularly beneficial in the context of acute hypovolemia, such as hemorrhagic shock, to maintain tissue perfusion and prevent end-organ injury. Moreover, HS decreases cellular oedema and may facilitate primary facial closure by reducing abdominal pressure and decreasing tension on the fascial edges. By drawing fluid out of the interstitial spaces, hypertonic saline helps in managing difficult closures, especially in cases of trauma or sepsis where tissue oedema may be particularly prominent. These changes have beneficial effects on the microcirculation and endothelial function, potentially increasing tissue oxygenation and reducing related complications such as abdominal compartment syndrome (ACS), wound dehiscence or incisional hernia. However, HS is also fraught with risks, such as hypernatremia, which is associated with its own complications, including seizures and renal insufficiency. Moreover, the direct evidence supporting its routine use specifically for aiding fascial closure in emergency laparotomy is somewhat limited and largely anecdotal, with most of the data coming from case reports, small studies, or extrapolation from its use in differing contexts such as cerebral oedema and intracranial pressure management [4,5].

In trauma patients, particularly those who are undertaking a DCL, the benefits of HS, such as reduced fluid requirements and enhanced capillary function, must be weighed against the potential risks of hypernatremia. Despite the potential of HS, the uniform development of hypernatremia in clinical practice renders its routine use quite controversial [6].

This study evaluates the efficacy and safety of hypertonic saline in comparison with isotonic fluids in patients subjected to a DCL. Outcomes that will be observed include primary fascial closure rates, mean time to fascial closure, mortality, incidence of hypernatremia, renal dysfunction (evidenced by rise in creatinine or the need for inpatient renal dialysis), 48-hour fluid totals, need for blood transfusion and ICU length of stay. A systematic review of such outcomes will establish whether or not the theoretical advantages of HS contribute to real clinical improvements.

The findings from this meta-analysis may have major implications for clinical practice by influencing the fluid resuscitation protocols under development in trauma surgery. As debates about the use of hypertonic saline in trauma care continue, this study will provide critical insight into whether HS should be widely adopted or reserved for specific situations where its benefits clearly outweigh the risks.

## Review

Methods

The systematic review and meta-analysis were conducted in accordance to the Preferred Reporting Items for Systematic Reviews and Meta-Analysis (PRISMA) statement standards [7].

Eligibility Criteria

All randomized control trials, non-randomized control trials, observational, and cohort studies looking at infusion of hypertonic saline in the management of emergency laparotomies were included. Hypertonic saline was the intervention of interest, while isotonic fluids were the comparator. All patients were included irrespective of age, gender and comorbidity status. Emergency laparotomies indicated by both trauma and sepsis were included. Case series with less than five patients were excluded, as well as articles not reported in English.

Primary and Secondary Outcome Measures

The primary outcome measures were early primary fascial closure (EPFC) rates, mean time to fascial closure, peak sodium (Na) concentration, and peak creatinine (Cr) concentration. Secondary outcomes included mean 48-hour fluid totals, need for blood transfusion, need for inpatient renal replacement therapy (RRT), and 28-day mortality.

Literature search strategy

Two authors, Neil Muscat (NM) and Shaneel Shah (SS), independently searched the electronic databases of MEDLINE, EMBASE, CINAHL, Google Scholar, Pubmed, and the Cochrane Central Register of Controlled Trials (CENTRAL). The last search was conducted on the 14th of June 2024. The search strategy utilized the thesaurus headings, search operators, and limits of each of the above databases. Search terms utilized for our intervention of interest involved "hypertonic saline", "laparotomy", "trauma", "primary", "sepsis", "sodium", "mortality", and "fascial closure". All terms were merged utilizing adjuncts of "or" and "and".

Selection of Studies

Titles and abstracts of articles retrieved from the literature search were independently reviewed by two authors, NM and SS. Articles which met the eligibility criteria had their full texts retrieved and reviewed. In the case of discrepancies, an independent third author, Neill Zammit (NZ), was obtained for review.

Data Extraction and Management

Microsoft Excel (Microsoft, Redmond, Washington) was used to create a data extraction table in line with Cochrane's data collection form for intervention reviews. The table was then pilot-tested in randomly selected articles and adjusted accordingly. It included study-related data: study name, year of publication, study design, number of patients in the intervention and control group and primary as well as secondary outcome measures as highlighted previously. Two authors, NM and NZ, collected and recorded the results, with any disagreements being resolved via discussion.

Data Synthesis

OpenMeta software was used for data synthesis. The extracted data was entered into the software by author NM. The analysis involved utilizing a random effects model with a generic inverse variance function being applied to account for cases of moderate to high heterogeneity. All results were reported on forest plots with 95% confidence intervals (CIs). For continuous data, a mean difference analysis was applied. Studies presenting continuous variable data as medians underwent conversion to a mean format in accordance with the formulae recommended by Luo et al. and Shi et al. [8,9]. For dichotomous data, an odds ratio (OR) was used, where the OR is the odds of an event happening in the HS group compared to the isotonic fluids group.

Assessment of Heterogeneity

Heterogeneity was assessed using the Cochrane Q test (X2), and the authors aimed to further quantify the inconsistency between studies by calculation of the I2 value. Studies with an I2 score between 0%-25% were deemed to have low heterogeneity, between 25-75% was considered moderate and 75-100% was associated with considerable heterogeneity.

Methodological Quality and Risk of Bias Assessment

Two authors, NM and NZ, independently assessed the methodological quality, as well as the risk of bias for articles that met the eligibility criteria. For randomized control trials, Cochrane's collaboration tool was used. Domains included in the assessment were selection, performance, detection, attrition, and reporting bias, as well as other sources. This allowed a classification into groups of either low, unclear or high bias. For observational studies, the Newcastle-Ottawa Scale [10] was used to assess the methodological quality with domains assessing selection, comparability and exposure. The scale uses a scoring system with a maximum total score of nine stars for each study. An independent third author (SS) was used to resolve any disagreements.

Results

**Figure 1:**
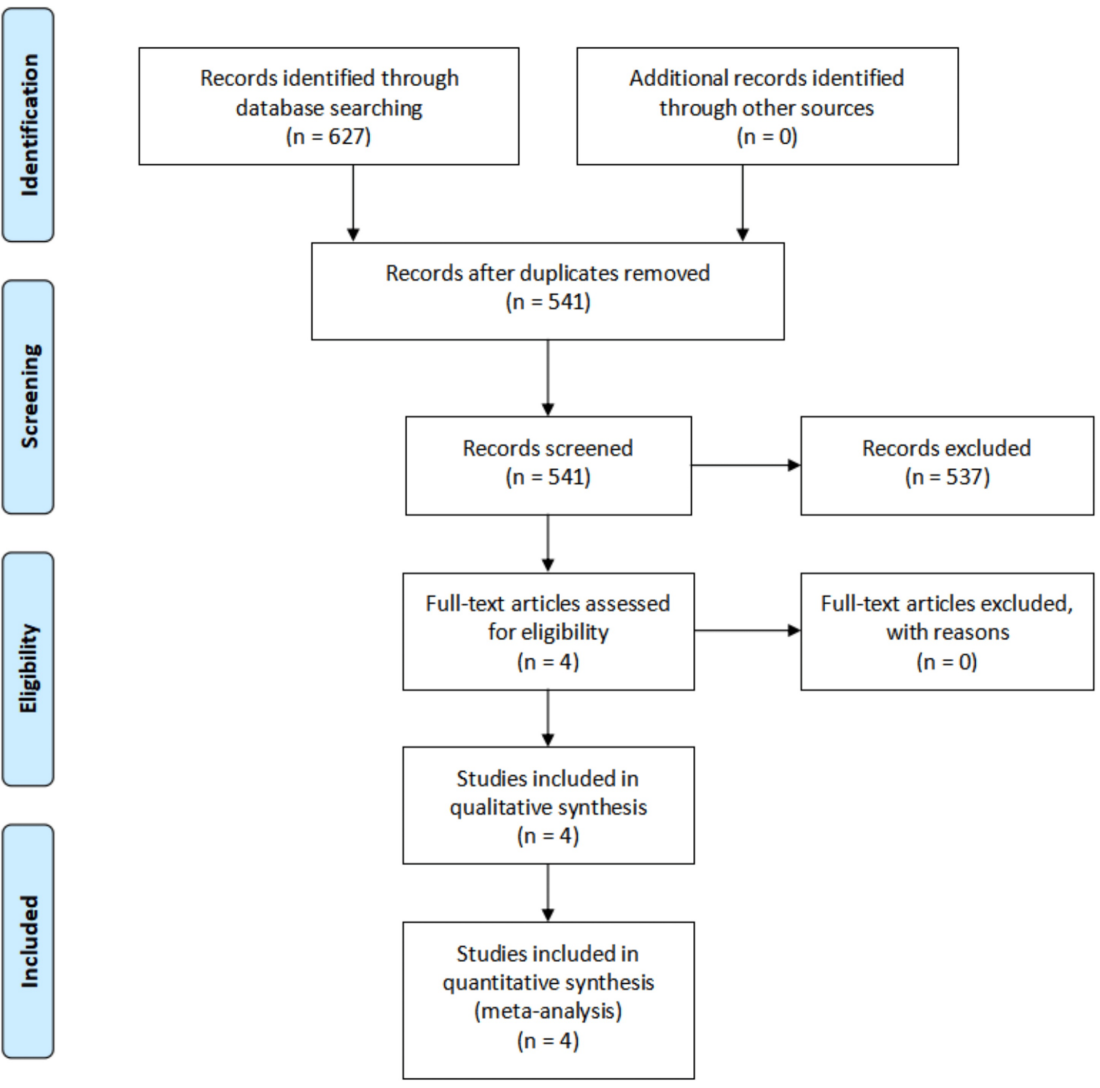
PRISMA flow diagram detailing the search and selection processes applied during the literature review PRISMA - Preferred Reporting Items for Systematic Reviews and Meta-Analysis

The search and selection process applied during this systematic review followed PRISMA guidelines (Figure 1).

Primary Outcomes

Early primary fascial closure (EPFC) rate: Harvin et al. [11] reported the highest PFC rate (100%) with HS, showing significant benefits in closure rate, while Loftus et al. [5] evidenced a slightly lower PFC rate with HS (92%), though it was not statistically significant. García et al. [12] found no significant difference between HS and isotonic fluids, with PFC rates of 79.2% and 70.8%, respectively, leading to their conclusion against HS use.

**Figure 2:**
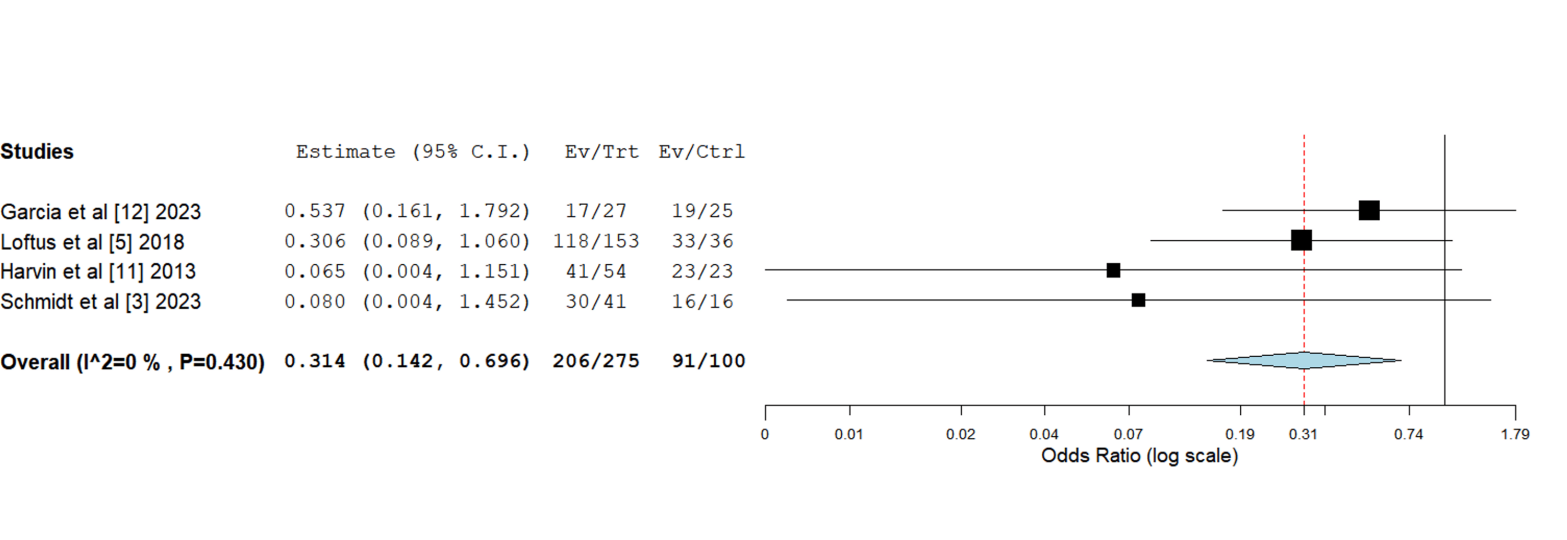
A forest plot depicting rates of early primary fascial closure which were found to be significantly higher with the hypertonic saline group compared to the isotonic fluids group Odds ratio 0.314 (0.142, 0.696); p-value 0.004

Schmidt et al. [3] evidenced intermediate outcomes with an 80.7% PFC rate in the HTS group, reporting a milder benefit compared to Harvin et al.'s study. On meta-analysis of these studies and odds ratio assessment utilizing a binary fixed-effects model with a generic inverse variance function to account for heterogeneity, these differences in outcomes were found to be statistically significant, favouring the use of HS in achieving EPFC (Figure 2).

**Figure 3:**
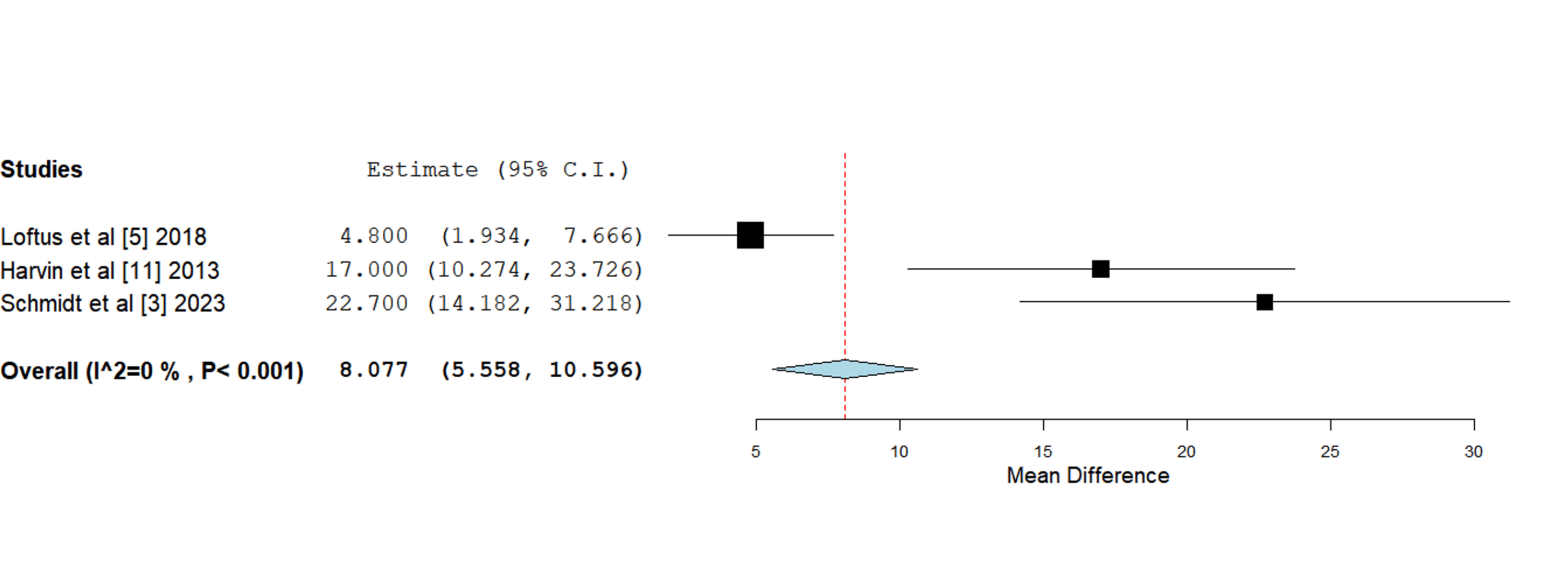
Mean time to primary fascial closure Mean difference 8.007 (5.558, 10.596), standard error 0.406, p-value <0.001

Mean time to primary fascial closure: García et al. [12] did not report a mean time to PFC in their study. The studies conducted by Harvin [11] and Schmidt [3] both evidenced a substantial reduction in the mean time to PFC with HS, with times of 33 hours and 36.4 hours, respectively, compared to the standard isotonic fluids group, which reported respective mean times of 50 hours and 59.1 hours. Loftus et al. [5] reported comparatively less pronounced differences, with the HS group achieving PFC in 40 hours compared to the standard isotonic fluid group, reporting a mean time of 55 hours. Meta-analysis of this data utilizing mean difference analysis evidenced a statistically significant shorter mean duration to primary fascial closure with the use of hypertonic saline (Figure 3).

Peak sodium (Na) concentration: All four studies consistently reported higher peak sodium concentrations in the HS groups compared to the standard isotonic fluids groups. The highest peak sodium concentration was reported by Loftus et al. [5] (150 mEq/L in the HS group), followed by García et al. (149 mEq/L) [12]. Schmidt et al. [3] and Harvin et al. [11] reported slightly lower peak sodium levels in the HS group but still higher than their respective control groups.

**Figure 4:**
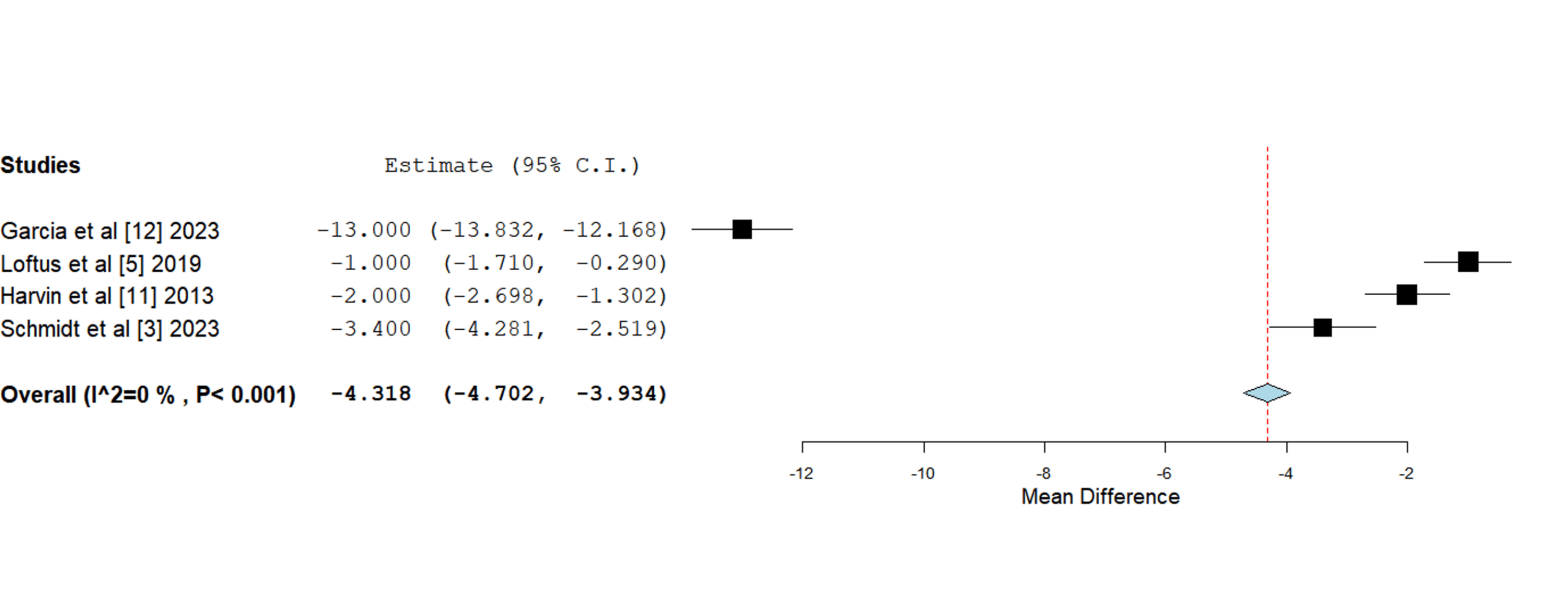
Forest plot depicting mean difference analysis of peak sodium concentrations between control and intervention groups Mean difference -4.318 (-4.702, -3.934), standard error 0.196, p-value <0.001

Across the studies, while elevated sodium levels were noted, they were always observed within a manageable range, and no severe adverse effects related to hypernatremia were reported, suggesting that with proper monitoring, the use of HS is generally safe. This comparison highlights the consistent effect of HS on raising peak sodium concentrations across different studies, with varying degrees of elevation, all of which remained within a clinically acceptable range when monitored closely. On mean difference analysis utilizing a continuous fixed-effects model and inverse variance function, the difference in peak sodium concentrations between intervention and control fluids was found to be statistically significant (Figure 4).

**Figure 5:**
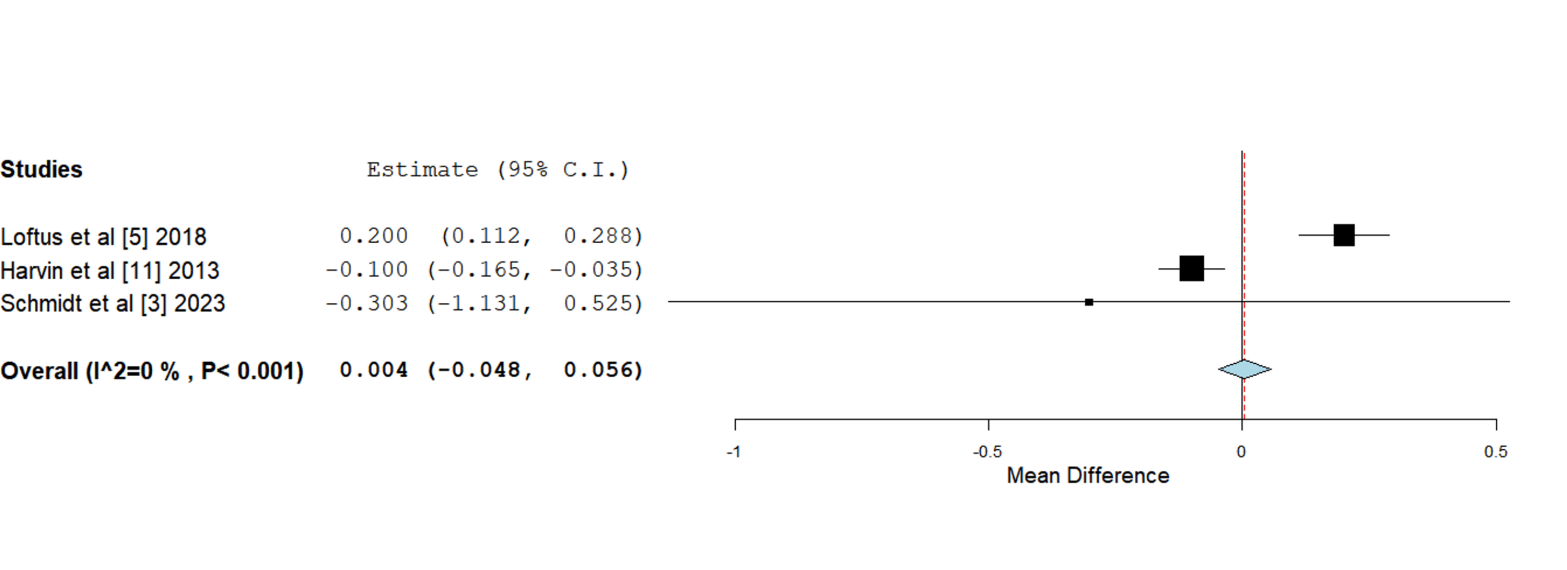
Forest plot depicting mean difference analysis of peak creatinine concentration between control and intervention groups Mean difference 0.004 (-0.048, 0.056), standard error 0.027, p-value 0.882

Peak creatinine (Cr) concentration: All four studies observed a very mild elevation in peak creatinine levels in the HS groups compared to their respective control groups. However, the differences were found to be statistically insignificant and did not translate into a higher incidence of kidney injury or the need for renal replacement therapy. The data pertaining to serum creatinine rise reported by García et al. [12] was presented as differing grades of severity ranging from a rise of 50% to 200%, which excluded its participation in formal quantitative analysis. The data from the included studies was subjected to a mean difference analysis through a continuous fixed-effects model. Overall, the rise in creatinine associated with the use of HS was determined to be statistically insignificant (Figure 5).

Secondary Outcomes

**Figure 6:**
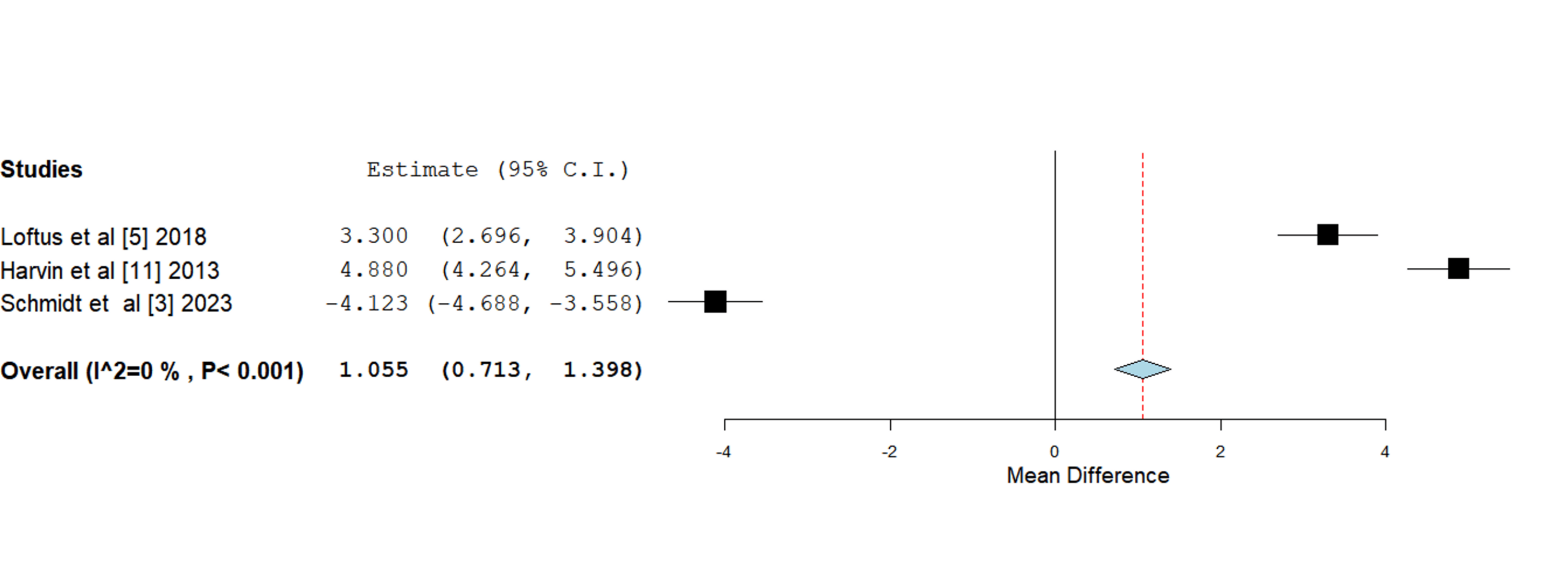
Forest plot depicting mean difference analysis of mean 48-hour fluid totals between control and intervention groups Mean difference 1.055 (0.713, 1.398), standard error 0.175, p-value <0.001

Mean 48-hour fluid totals: All included studies consistently reported that the use of HS results in significantly lower 48-hour fluid totals compared to standard or isotonic fluids. Harvin et al. [11] reported the lowest fluid totals in the HS group (6290 mL), which was nearly half of that required by the isotonic fluids group. Loftus et al. [5] and García et al. [12] reported slightly higher 48-hour fluid totals in their HS groups (7100 mL and 7800 mL, respectively) compared to Harvin et al. [11]; however, these were still significantly lower than the totals for the standard fluids groups. The study conducted by Schmidt et al. [3] arrived at a similar conclusion, with the HS group requiring 8,086 mL of fluid over 48 hours, compared to 12,987 mL in the isotonic fluids group. The data reported by García et al. [12] was reported in a way that excluded it from a meta-analysis. Upon further statistical analysis, the 48-hour fluid mean difference seen with the use of HS when compared to isotonic fluids was found to be statistically significant (Figure 6).

**Figure 7:**
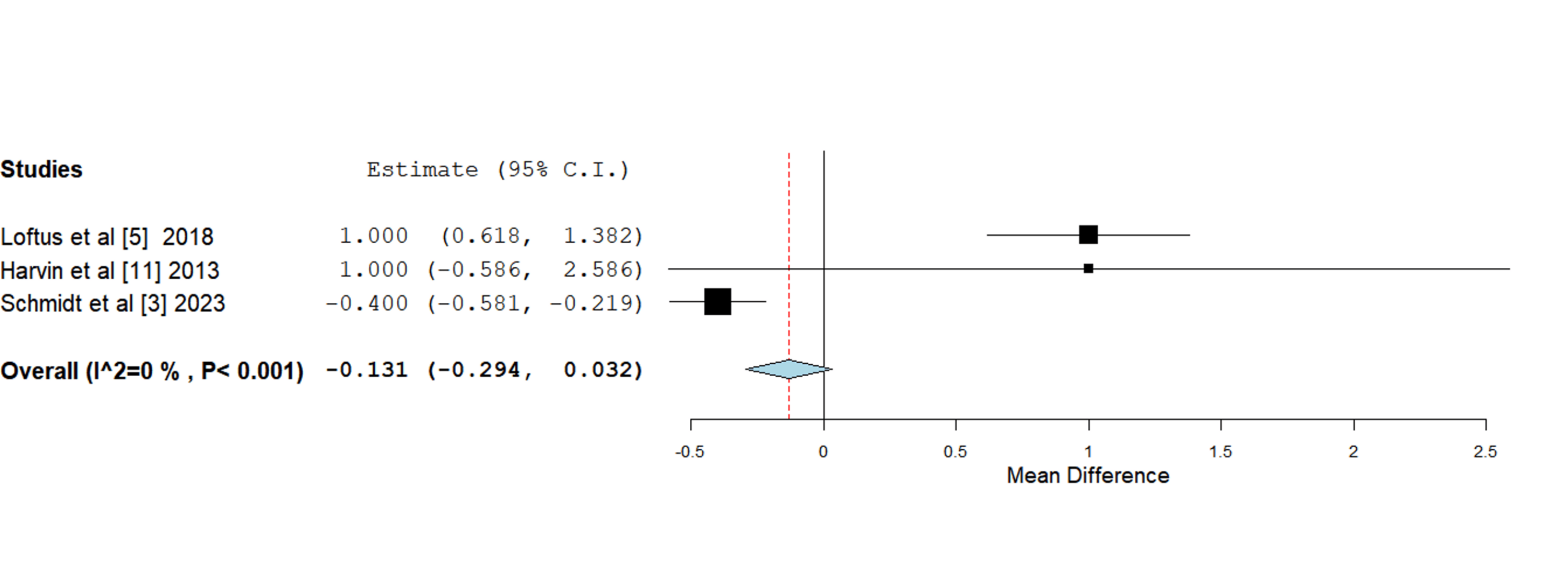
Forest plot depicting mean difference analysis of the need for blood transfusion between control and intervention groups Mean difference -0.131 (-0.294, 0.032), standard error 0.083 , p-value <0.116

Need for blood transfusion: Across all included studies, the HS groups required slightly fewer red blood cell (RBC) units compared to the standard or isotonic fluids groups. However, the differences in the need for blood transfusions were minor and not statistically significant in the mean difference analysis. Once again, the data reported by García et al. [12] was reported in a way that excluded it from formal statistical analysis (Figure 7).

**Figure 8:**
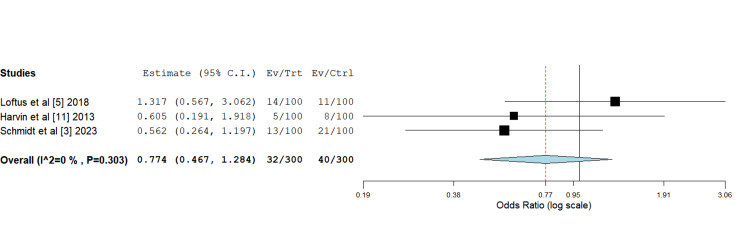
Forest plot depicting odds ratio analysis of the need for renal replacement therapy between control and intervention groups Odds ratio 0.774 (0.467, 1.284), standard error 0.258, P-value = 0.332

Need for inpatient renal replacement therapy (RRT): García et al. [12] did not report any data pertaining to this outcome. Across the remaining included studies, there was a consistent trend of a slightly higher percentage of patients in the HS groups requiring renal replacement therapy compared to the standard isotonic fluids groups. However, on odds ratio analysis utilizing a binary fixed-effects model, the differences were small and not statistically significant (Figure 8).

**Figure 9:**
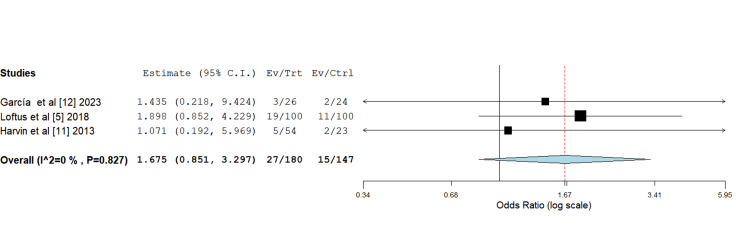
Forest plot depicting odds ratio analysis of 28-day mortality rates between control and intervention groups Odds ratio 1.675 (0.851,3.297), standard error 0.346, p-value 0.136

Twenty-eight-day mortality: Schmidt et al. [3] did not report any outcome pertaining to mortality. Across the rest of the included studies, the 28-day mortality rates between the HS and control groups were very similar, with no statistically significant differences. Harvin et al. [11] evidenced comparable mortality rates between the HS and standard fluids groups, while Loftus et al. [5] reported a slightly higher, statistically insignificant mortality rate for the standard fluids group. García et al. [12] reported the lowest 28-day mortality rates among the studies, with a minor, non-significant difference favouring the HS group. Overall, the difference in 28-day mortality between the control and intervention groups was found to be statistically insignificant in statistical analysis (Figure 9).

Miscellaneous Outcomes

Harvin et al. [11] and García et al. [12] reported outcomes pertaining to ICU-free days. The overall trend across these studies suggests that HS may contribute to more ICU-free days compared to isotonic fluids, potentially due to quicker recovery, fewer complications, or both. This effect was statistically significant with regard to Harvin et al.'s study [11], with the control group experiencing 15 ICU-free days compared to 23 days experienced by the HS group.

The use of HS and its effect on mean ventilator-free days was studied by three of the four included studies. Loftus et al. [5] reported one less ventilator-free day with the HS group, Harvin et al. [11] reported four additional ventilator-free days, whilst Schmidt et al. [3] reported a reduction of 1.79 ventilator-free days.

The hospital length of stay experienced by the control and intervention groups was compared by Loftus et al. [5] and Schmidt et al. [3]. Schmidt et al. reported that patients receiving HS spent 3.8 fewer days in hospital, while Loftus et al. reported a 0.3 day longer stay in hospital with the HS group.

Loftus et al. and Schmidt et al. also reported data concerning differences in arterial pH in patients receiving isotonic fluids when compared to HS. In the study conducted by Loftus et al., the mean pH for the isotonic fluids group was 7.38 when compared to the mean pH of 7.34 experienced by the HS group. Similarly, Schmidt et al. reported a mean pH of 7.49 for the control fluids group compared to a pH of 7.48 in the HS group [3,5].

The methodological quality review was assessed with the Cochrane collaboration tool for all randomized control trials (Table 1), and the Newcastle-Ottawa scale [10] was instigated for the assessment of all observational studies (Tables 2,3). 

**Table 1:** Assessment of risk bias for the randomized control trial by García et al., utilizing the Cochrane collaboration tool García et al. [12]

Selection bias random sequence generation	Low	Computer generated randomization
Selection bias: allocation concealment	Low	Hidden from patients
Reporting bias: selective reporting	Low	All outcomes presented
Other bias: other sources of bias	Low	No other bias detected
Performance bias: double blinding (participants and personnel)	Unclear	Both assessors and patients were blinded
Detection bias: blinding (outcome assessment)	Low	Assessors blinded
Attrition bias: incomplete outcome data	Low	Four patients from the control group, one patient from intervention group

**Table 2:** Newcastle-Ottawa Scale (NOS) to assess the quality of non randomized studies

Study	Selection	Comparability	Exposure
Loftus et al. [5]	*	-	*
Harvin et al. [11]	*	-	*
Schmidt et al. [3]	*	*	*

**Table 3:** Amalgamation of study data including author name, year of publication, type of study and number of patients HTS - hypertonic saline

Author Name	Year of publication	Type of study	Total Number of patients	Number in control group	Number in intervention group (HTS)
Harvin et al. [11]	2013	Retrospective cohort	77	54	23
Loftus et al. [5]	2018	Retrospective cohort	189	153	36
García et al. [12]	2023	Randomized controlled trial	52	27	25
Schmidt et al. [3]	2023	Retrospective cohort	57	41	16

Discussion

This systematic review and meta-analysis investigate the efficacy and safety of the use of hypertonic saline in damage control laparotomy (DCL) for trauma and sepsis, considering its potentially beneficial application with regard to primary fascial closure and fluid management. Here, we discuss the methodologies and results of these studies, as well as the implications of their findings.

The primary goal in trauma cases is to control bleeding, prevent contamination from bowel perforations, and stabilize the patient for further treatment. For patients with sepsis, particularly abdominal sepsis, an emergency laparotomy may be required to control the source of infection. This might involve procedures such as resection of necrotic bowel, drainage of abscesses, or repair of perforated peptic ulcers. The aim is to remove or isolate the infectious source, thereby reducing the systemic inflammatory response and preventing further deterioration of the patient's condition. Laparotomy for trauma or for intra-abdominal sepsis shares similar underlying principles of damage control and restoration [13].

Outcomes of emergency laparotomies vary significantly depending on factors such as the patient's overall comorbidity status, the extent of injuries or infection, and the timeliness of the intervention. Fascial closure is a critical step in emergency laparotomy, essential for restoring the integrity of the abdominal wall following operative intervention. The primary goal is to re-approximate the fascial edges to prevent complications and ultimately reduce morbidity and mortality, especially in cases of severe trauma or advanced sepsis. Complications of poor fascial closure include wound dehiscence, incisional hernia, superficial and deep wound infections, protracted hospital stay and delayed recovery [14].

The choice of technique for fascial closure can vary depending on the patient's condition and the extent of the surgery. Historically, surgeons have preferred primary closure using a mass closure technique, which boasts a strong, durable repair. Emerging evidence supports the use of the small bites closure technique. This method uses less suture material, which has the theoretical benefits of adequate tension without compromising blood flow to the tissue. This helps reduce the risk of ischemia and promotes better healing [15].

In cases where the abdominal wall cannot be closed primarily due to swelling, contamination, or other factors, a temporary closure technique, such as using a vacuum-assisted closure (VAC) device or a mesh, may be employed. This allows for delayed primary closure once the patient is more stable and the abdominal contents have reduced in size [16,17].

In the single trauma centre, double-blinded randomized controlled trial by García et al. [12], 52 patients with abdominal injuries requiring DCL were evaluated. Patients were randomly divided into groups receiving 3% HS or NS given at a constant rate over 72 hours post-operatively. The primary outcome measure in this study was the rate of primary fascial closure within seven days. The trial was stopped early, citing futility, as the interim analyses indicated that the expected benefits of HS were not materializing. This study's robust design is somewhat undermined by the small sample size and early termination, thus limiting generalizability.

Loftus et al. [5] supplemented this research with a multicenter retrospective review of 189 patients at various trauma centres. This included a larger and more varied cohort of patients receiving either HS or NS after DCL. The authors retained primary fascial closure as a primary outcome measure but extended the follow-up period to six months to establish data on long-term outcomes, including mortality and organ dysfunction. This study's sphere of relevance thus extends to the sustained after-effects of HS use in trauma care.

Harvin et al. [11] designed a retrospective cohort study and used multi-hospital trauma registries in a sample size of 77 patients, comparing HS recipients versus NS recipients in the ICU following DCL. The primary outcome measures included primary fascial closure, mortality, and renal function. This study, similar to the research conducted by Loftus et al. [5] and Schmidt et al. [3], is retrospective in nature and hence subject to all biases that occur in such studies, especially selection bias and confounding factors.

The retrospective study conducted by Schmidt et al. [3] analyzed data concerning patients who had penetrating abdominal trauma. This was a single-centre study undertaken to assess the efficacy of HS in increasing the primary fascial closure rate with additional monitoring for electrolyte disturbances and renal dysfunction. This study provides specific insight into penetrating trauma; however, overall, it is limited with respect to the small sample size for the robustness of its conclusions.

Early primary fascial closure rate was the key primary outcome evaluated in this meta-analysis, with results constantly advocating for the use of HS in this context. Harvin et al. [11] recorded a 100% EPFC rate, which was the highest rate among all the studies included in this meta-analysis. Schmidt et al. [3] reported an EPFC of 80.7%, while Loftus et al. [5] evidenced a similarly high EPFC rate of 92%, although this did not reach statistical significance when compared to the control group.

Perhaps the reason for the ability of HS to facilitate improvement in EPFC rates is its hyperosmolar properties, which facilitate the reduction of tissue oedema and intra-abdominal pressure hence, in effect, achieving the closure of the fascia. HS pulls fluid from the interstitial spaces, reduces the tension of the fascial edges, and therefore increases the chance of successful fascial closure in the early postoperative period. This finding may be important in the context of a trauma patient, for whom rapid, effective closure is of utmost importance in minimizing complications such as abdominal compartment syndrome, infection, need for further surgery and subsequent wound dehiscence [18].

One must consider the potential risks of such therapy, including hypernatremia and renal dysfunction. However, in general, these were easily managed and, again, did not show any significant adverse effect throughout the studies conducted. Nonetheless, this calls for very careful monitoring and safe use in patients with a background of chronic renal impairment.

The side effects of HS were evaluated based on peak sodium and creatinine values and the need for inpatient RRT. In all studies, the peak sodium value was reported to be higher in the HTS group. The maximum peak sodium reported by Loftus et al. [5] was as high as 150 mEq/L. Although peak sodium levels were observed to be higher, no severe hypernatremia-related side effects were reported, indicating that it is safe to use HS if sodium values are monitored continuously.

The elevation of creatinine levels as a marker of renal function showed only mild elevations in the HS groups, with no statistical difference between intervention and control groups on a meta-analysis of the data. Similarly, there was also a trend for increased demand for RRT in the HS groups, yet without statistical significance. This suggests that the use of HS is generally safe with respect to renal function and does not markedly increase the likelihood of requiring RRT. However, due to the small differences observed, it remains important to monitor renal function closely in patients receiving HS, particularly in those who may already be at risk of renal complications.

A significant advantage of HS demonstrated in all included studies was the fact that it decreases overall fluid requirements. The intervention group required significantly less fluid over the 48-hour period compared to the control group. This reduction in fluid volume is attributed to the hypertonic nature of the saline, which helps to draw fluid into the vascular space, reducing the overall need for additional resuscitative fluids. Clinically, this can be of considerable significance due to the possible reduction of complications arising from fluid overload, such as pulmonary oedema, abdominal compartment syndrome and acute coronary syndromes. The lower total fluid requirements with HS use highlight its potential benefit in managing patients undergoing damage control laparotomy, particularly in those at risk for fluid overload [19].

There were no significant differences between the HS and control groups with regard to the need for blood transfusions. Although the need for transfusions tended to be less when HS was given, these differences were not statistically significant, which means that HS may lack clinical significance for end-points such as bleeding or the need for transfusion in the acute setting. While there is a trend toward slightly lower transfusion requirements in the HS groups, this difference is not clinically significant. The consistent finding suggests that while HS is effective in reducing fluid requirements, it does not have a marked impact on the need for blood products, implying that blood transfusion needs are more closely related to the extent of blood loss and overall injury severity rather than the type of fluid resuscitation used.

The 28-day mortality rate did not significantly differ between the HS-treated and control groups in the reviewed studies. This suggests that, although HS may provide for better fluid management with fascial closure, this does not seem to have an association or effect on overall survival in the short term.

This systematic review and meta-analysis were limited by differing selection criteria set by each of the included studies. Although each study briefly discussed precautions taken to account for variable patient ages, medical comorbidities and severity of trauma sustained, there was no standardized method applied throughout all the included studies to fully take this into account, thus limiting the generalizability of the results. This highlights the need to investigate this topic within the frame of a randomized-control trial with strict inclusion and exclusion criteria and larger sample sizes to produce reliable results. 

## Conclusions

The authors present a systematic review and meta-analysis investigating the use of HS in DCL. These findings imply that although HS may have statistically significant impacts on specific outcome measures, such as fluid balance and primary fascial closure, this does not contribute to broader patient recovery as measured by short-term mortality and ICU or hospital stay. As such, while the theoretical benefits are recognized, its use should typically be considered on a case-by-case basis, with the decision being influenced by the overall clinical condition of the patient and the judgement of the surgical team. These conclusions and discussed limitations strongly frame the need for randomized controlled trials with larger sample sizes conducted in more heterogeneous patient populations and stricter selection criteria to better define the role of hypertonic fluids in trauma care and establish more definitive guidelines for their use.
